# Ultrafast Quenching of Excitons in the Zn_x_Cd_1−x_S/ZnS Quantum Dots Doped with Mn^2+^ through Charge Transfer Intermediates Results in Manganese Luminescence

**DOI:** 10.3390/nano11113007

**Published:** 2021-11-09

**Authors:** Dmitry Cherepanov, Andrei Kostrov, Fedor Gostev, Ivan Shelaev, Mikhail Motyakin, Sergei Kochev, Yuriy Kabachii, Victor Nadtochenko

**Affiliations:** 1 N.N. Semenov Federal Research Center for Chemical Physics, RAS, Kosygin St. 4, 119991 Moscow, Russia; tscherepanov@gmail.com (D.C.); andreikostrov@rambler.ru (A.K.); boatsween@yandex.ru (F.G.); shelaev@bk.ru (I.S.); motyakin@hotmail.com (M.M.); 2 A.N. Nesmeyanov Institute of Organoelement Compounds, RAS, Vavilov St. 28, 119991 Moscow, Russia; kochew@ineos.ac.ru (S.K.); kabachi@ineos.ac.ru (Y.K.); 3 Department of Chemistry, Lomonosov Moscow State University, Leninskiye Gory 1–3, 119991 Moscow, Russia

**Keywords:** quantum dot, femtosecond transient absorption, stark shift, exciton

## Abstract

For the first time, a specific time-delayed peak was registered in the femtosecond transient absorption (TA) spectra of Zn_x_Cd_1−x_S/ZnS (x~0.5) alloy quantum dots (QDs) doped with Mn^2+^, which was interpreted as the electrochromic Stark shift of the band-edge exciton. The time-delayed rise and decay kinetics of the Stark peak in the manganese-doped QDs significantly distinguish it from the kinetics of the Stark peak caused by exciton–exciton interaction in the undoped QDs. The Stark shift in the Mn^2+^-doped QDs developed at a 1 ps time delay in contrast to the instantaneous appearance of the Stark shift in the undoped QDs. Simultaneously with the development of the Stark peak in the Mn^2+^-doped QDs, stimulated emission corresponding to ^4^T_1_-^6^A_1_ Mn^2+^ transition was detected in the subpicosecond time domain. The time-delayed Stark peak in the Mn^2+^-doped QDs, associated with the development of an electric field in QDs, indicates the appearance of charge transfer intermediates in the process of exciton quenching by manganese ions, leading to the ultrafast Mn^2+^ excitation. The usually considered mechanism of the nonradiative energy transfer from an exciton to Mn^2+^ does not imply the development of an electric field in a QD. Femtosecond TA data were analyzed using a combination of empirical and computational methods. A kinetic scheme of charge transfer processes is proposed to explain the excitation of Mn^2+^. The kinetic scheme includes the reduction of Mn^2+^ by a 1Se electron and the subsequent oxidation of Mn^1+^ with a hole, leading to the formation of an excited state of manganese.

## 1. Introduction

Doped quantum dots (QDs) are promising materials for different applications in photovoltaics, spintronics, bioimaging, photonics, and other modern technological applications [1,2,3,4,5,6,7,8,9,10,11,12,13,14,15,16,17,18,19]. Doping II-VI semiconductor QDs with Mn^2+^ ions makes it possible to obtain brightly luminescent nanomaterials, which are used in bioimaging, photocatalysis, and other applications [5,11,20,21,22]. The orange-red luminescence of Mn^2+^ in the 580–600 nm region associated with the ^4^T_1_-^6^A_1_ transition is induced in the QDs through an exciton absorption. The ^4^T_1_-^6^A_1_ transition is forbidden by the selection rule since a spin flip-flop is required, and it therefore has low oscillator strength [2,6,10,11,23]. At the same time, the Mn^2+^ ion effectively quenches the exciton, and the Mn^2+ 4^T_1_-^6^A_1_ luminescence quantum yield can reach ~45–65%, which means a high probability of energy transfer from the exciton to Mn^2+^ [24,25,26]. The mechanism of energy transfer from the exciton to the d^5^ levels of the Mn^2+^ ion remains controversial [10,11,12,15,19,21,23,27,28]. The excitation of Mn^2+^ can either be due to the energy transfer or charge transfer by the host or the surface ligand. The prevailing opinion in the literature is that the Mn^2+^ excitation is realized by nonradiative energy transfer processes Förster-, Dexter-, or Auger-like rather than charge transfer [10,12,16,23,27,29].

The magnetic ion Mn^2+^ has five electrons that half-fill the d^5^ levels. In II-VI semiconductors, the ground state Mn^2+^(d^5^) is characterized by a spin S = 5/2, an angular momentum L = 0, and a negligible spin-orbit splitting. The Mn^2+^(d^5^) as a magnetic impurity in a semiconductor is characterized by a strong exchange interaction of the d electrons of Mn^2+^(d^5^) with electrons in the conduction bands (s-band) and holes from the valence band (p-band). The exchange coupling between the sp-electrons of exciton and the d-electrons of Mn^2+^(d^5^) ions (sp-d mixing) determines the formation of the exciton magnetic polaron [4,9,12,18]. It was suggested that the sp–d mixing and direct exchange interactions could be responsible for the energy transfer between exciton and Mn^2+^ [23,30]. This mechanism implies that non-radiative recombination remains efficient even when the bandgap *E*g substantially exceeds the energy of the ^6^A_1_-^4^T_1_ transition, i.e., the energy transfer is directed into the upper Mn^2+^ excited states in that case. The Förster dipole–dipole mechanism, despite the forbidden transition for Mn^2+^, was also considered [27,29]. Y. Yang et al. suggested a two-step mechanism for energy transfer [31]. In core/shell CdS/ZnS QDs, the energy transfer from an exciton inside the CdS core occurs to a bound exciton around a Mn center. This is the rate-determining step, which is then proceeded by the Förster mechanism [31]. The second step is the energy transfer from the bound exciton to the d^5^ levels of Mn^2+^. It might be a dark exciton (triplet exciton) or an Auger transfer mechanism [31]. Two sequential charge transfer reactions between exciton and manganese dopant were suggested for the explanation of manganese excitation in Cd_x_Zn_1−x_Se (x = 0–0.2) [28]. In this system, a band near 2.5 eV was reported in the luminescence and TA spectra, and it has been attributed to the spin-allowed ^5^T_2_-^5^E transition of Mn^3+^. Based on this assignment of 2.5eV band, a scheme of Mn^2+^(^4^T_1_) excitation was proposed with Mn^3+^ as an intermediate appearing due to Mn^2+^ oxidation by the hole. The hole scavenging by Mn^2+^ and the excitation of the Mn^3+^(^5^T_2_) state occur with a characteristic time of ~200 fs. It was declared that the luminescent Mn^2+^(^4^T_1_) state arises from the Mn^3+^(^5^T_2_) state with a characteristic time of 300–800 ps [28].

Recently, we reported the stimulated emission of Mn^2+^ (^4^T_1_-^6^A_1_) registration in femtosecond TA spectra at subpicosecond time delays in Mn^2+^-doped QDs Zn_0.5_Cd_0.5_S in a short letter, but the mechanism of manganese excitation remained unclear [26]. It is noteworthy that the characteristic band near the 2.5 eV of Mn^3+^ (^5^T_2_-^5^E) transition was not observed in the Mn^2+^: Zn_0.5_Cd_0.5_S QDs [26]. Therefore, the purpose of this work is to carry out additional studies of the femtosecond kinetics of TA spectra of Mn^2+^ alloy-doped and undoped QDs Zn_0.5_ Cd_0.5_S in a spectral range of 390–700 nm. In the current work, we report for the first time that the TA spectra of the manganese-doped QDs revealed a time-delayed absorption peak appearing at the red side of the edge exciton. This specific peak can be attributed to an electrochromic Stark shift of the band-edge exciton. The kinetics of this absorption peak, related to the delayed Stark shift of the exciton band, can expose the dynamics of charge transfer intermediates in the process of Mn^2+^(d^5^) excitation. A global analysis of TA spectra dynamics, taking into account the dynamics of Stark shift, suggests the mechanism of manganese excitation through the charge transfer intermediates formation.

## 2. Materials and Methods

Chemicals. Zinc acetate dihydrate (Zn(OAc)_2_•2H_2_O, 98%), cyclohexane (99+%, for spectroscopy), 1-octadecene (ODE, 90%, tech.), manganese (II) chloride (MnCl_2_, anhydrous, 99%), tetramethylammonium hydroxide (TMAH, 25 wt% in methanol), CdO (99.5%), cadmium acetate dihydrate (Cd(OAc)_2_•2H_2_O, ≥98%), oleic acid (90%, tech.), stearic acid (HSt, 95%), oleylamine (OlAm, C18 content 80–90%), and stearic acid (HSt, 95%) were acquired from Merck (Merck KGaA, Darmstadt, Germany). Magnesium turnings (Mg, 99%) and sulfur (reagent grade, 100 mesh) were bought from Sigma-Aldrich (Sigma-Aldrich Chemie GmbH, Steinheim, Germany). Anhydrous methyl alcohol (MeOH) was prepared from chemically pure grade MeOH by boiling it with magnesium methylate. Manganese stearate (MnSt_2_) was synthesized from MnCl_2_.

Transmission electron microscopy (TEM) images were registered using a LEO 912 AB OMEGA (Karl Zeiss, (Jena, Germany)) microscope. The accelerating voltage was 100 kV. The particle diameters distribution in a group of 300 QDs was measured with ImageJ software.

Elemental microanalysis. An atomic absorption spectrometer with flame atomization KVANT-2AT (CORTEC, Moscow, Russia) was used to characterize the elemental composition of QDs. QDs were digested by acid mineralization (micro-Kjeldahl digestion) before injection in the atomic absorption spectrometer.

EPR spectra. X-band EPR spectra were recorded on a Bruker EMX (Karlsruhe, Germany) spectrometer operating at 9.7 GHz and 100 kHz magnetic field modulation. The samples were placed into a resonator of the spectrometer using 4 mm i.d. quartz tubes. The spectral acquisition was carried out with following parameters: QDs colloids in cyclohexane; temperature, 293 K; conversion time, 327.7 ms; time constant, 40.96 ms; modulation amplitude, 0.9 G; the number of points, 2048; microwave power, 6.5 mW; and sweep width 1200 G. WINEPR and SIMPHONIA (Bruker) programs were used for the mathematical treatment of EPR spectra.

Optical spectra. Absorption spectra were recorded with a Shimadzu 3600 spectrophotometer (Kyoto, Japan). Photoluminescence excitation (PLE) and photoluminescence (PL) experiments were performed on a Shimadzu RF-5301PC spectrofluorimeter at room temperature. Coumarin 6 in ethanol, rhodamine 6G, and rhodamine B in methanol in ethanol as a PL reference were used to measure the quantum yield (QY) [32]. Coumarin 6 was used for the estimation of QY of exciton luminescence in the range of ~430–460 nm, whereas Rhodamine B and Rhodamine 6G were used to estimate the QY of Mn^2+^ luminescence. Rhodamine B and rhodamine 6G were used to estimate the QY luminescence of Mn^2+^. Coumarin 6 was used to estimate the QY of exciton luminescence in the range of ~430–460 nm.

Femtosecond transient absorption (TA). TA spectra were measured by the broadband femtosecond pump-to-probe technique (Federal Research Center of Chemical Physics RAS, Moscow, Russia). The pump pulse was 30 fs centered, 360 nm, 40 nJ. The diameter of the pump spot was 300 µm, and the probe pulse was 120 µm. The pump–pulse operation frequency was 100 Hz. The sample was refreshed between incident laser pulses by a pump in a 500 μm cell. The polarization of the pump–probe was oriented at a magic angle of 54.7°. The solvent was cyclohexane. The temperature of the cell was 293 K. The measured spectra were corrected to account for the group delay dispersion of the supercontinuum by the procedure described in Supplementary Information (Section S1). Details of the setup are presented elsewhere [26,33,34].

## 3. Results and Discussion

### 3.1. QDs Characterization

Femtosecond transient spectroscopy experiments were carried out with two types of QDs. The quantum dots of the first type (QD-1) had the composition Zn_x_Cd_1−x_S, x = 0.5 with a diameter of 5.7 ± 0.9 nm and were not doped with Mn^2+^. The synthesis protocols, TEM images, and nanoparticle size distribution are shown in Figure S1. The second type of quantum dots (QD-2) had a composition core/shell Mn:Zn_x_Cd_1−x_S/ZnS, x = 0.46 with a diameter of 7.6 ± 0.9 nm and were doped with Mn^2+^. The Mn/Cd molar ratio was 0.06 mol%, which corresponds to the average number of Mn ions per quantum dot <μ_Mn_> = 7.8. The shell prevented Mn^2+^ ions from washing out from the QDs and provided a sufficiently high quantum yield (QY~0.6) of Mn^2+^ luminescence upon excitation at a wavelength of 360 nm.

**Figure 1:**
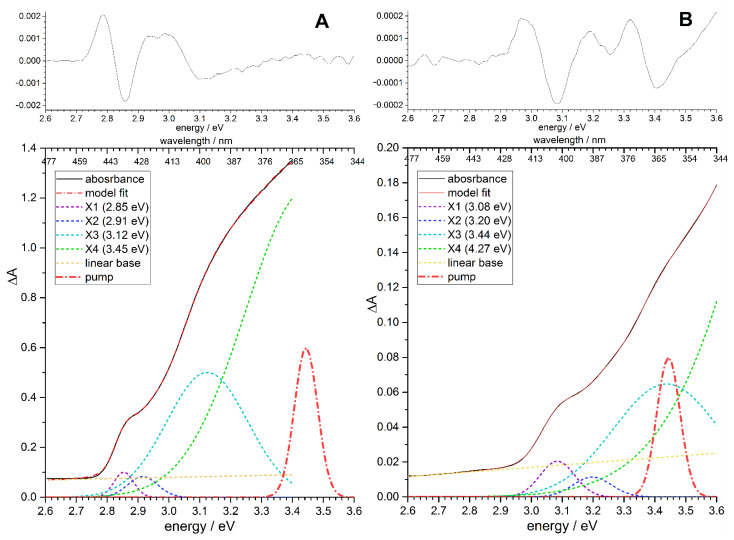
Absorption spectra of QD-1 (**A**) and QD-2 (**B**) quantum dots. Upper subplots show absorption spectra in the form of the second derivative. Lower subplots show absorption spectra and their decomposition into Gaussian peaks.

Figure 1 shows the absorption spectra of QD-1 and QD-2 quantum dots. A presentation of QDs spectra in the form of the second derivative made it possible to determine the positions of exciton peaks [35]. The absorption spectra were decomposed into four Gaussian peaks of the exciton bands (see Table 1).

**Figure 2:**
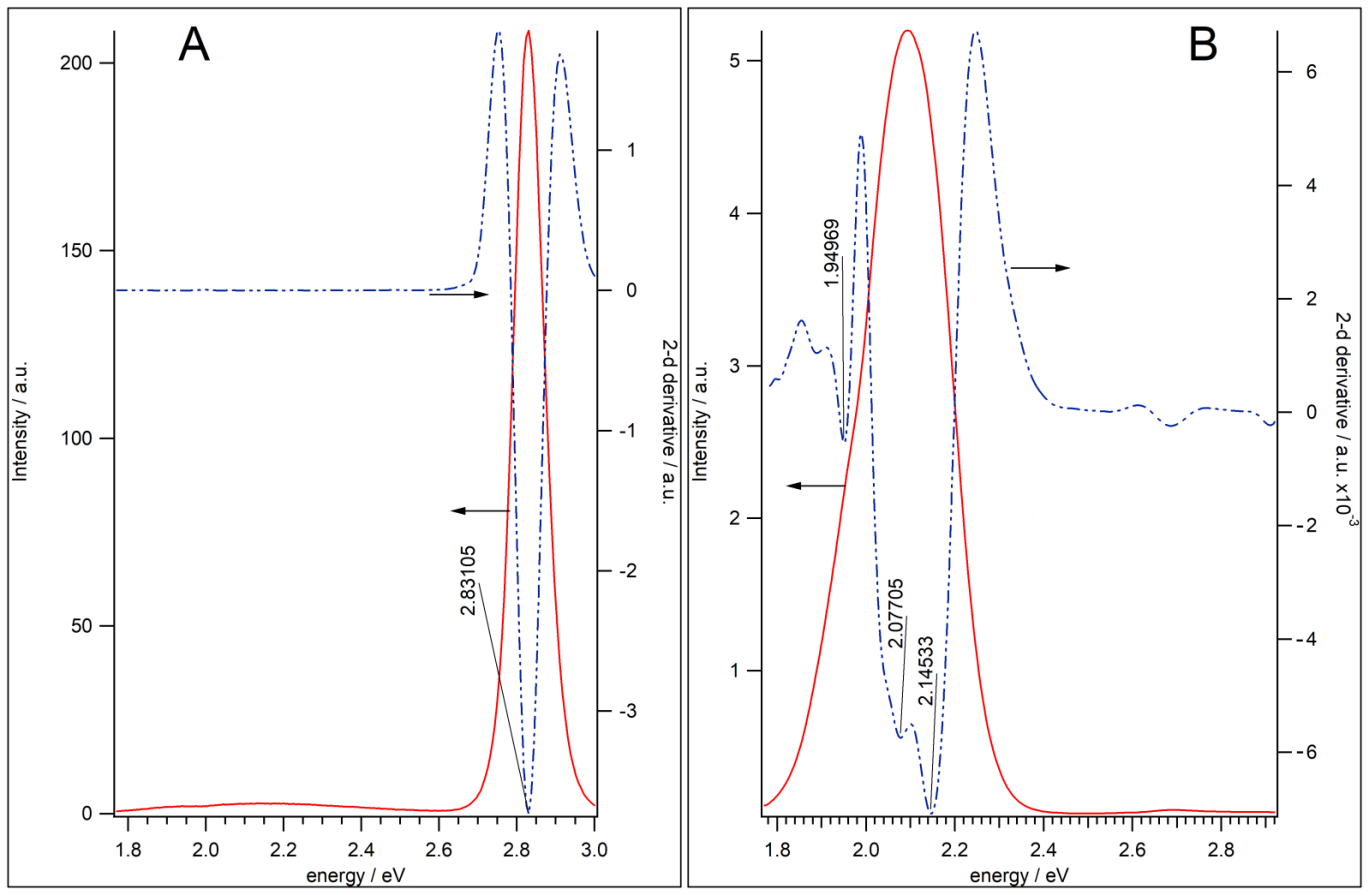
PL spectra of undoped QD-1 (**A**) and Mn^2+^-doped QD-2 (**B**) samples. The PL spectra (red solid lines, left y-axis) are compared with the second derivatives of spectra (blue dash-dotted line, right y-axis).

Figure 2 demonstrates the PL spectra of the QD-1 and QD-2 samples. The undoped QD-1 sample reveals an intense PL peak at 2.831 eV (438 nm) associated with the band-edge exciton luminescence, and a very weak broad band at 2.127 eV (583 nm) fwhm = 360 meV (~130 nm) attributed to traps (Figure 2A). The PL amplitude of the traps band was 80 times less than the amplitude of the exciton peak. Figure 2B demonstrates that in the doped QD-2 Mn^2+^:Zn_0.46_Cd_0.54_S/ZnS sample, exciton emission was suppressed and the band close to ~2.1 eV, associated with the PL of Mn^2+^ ions, dominated. This band was inhomogeneous, and several peaks can be distinguished corresponding to the minima of the second derivative [35]. The PLE spectrum of Mn^2+^ is similar to the absorption spectrum of QD-2 Mn^2+^:Zn_0.46_Cd_0.54_S/ZnS sample in the region close to the band-edge exciton (Figure S2). This indicates that the Mn^2+^ luminescence appears to be due to exciton absorption in the Zn_0.46_Cd_0.54_S/ZnS QD, and the quantum yield of ~60% indicates high efficiency of the energy transfer from exciton to d5 levels of manganese ion.

Electron paramagnetic resonance (EPR) spectroscopy can provide insight into crystal field features and the distribution of dopant ions inside QD-2 nanocrystals.

**Figure 3:**
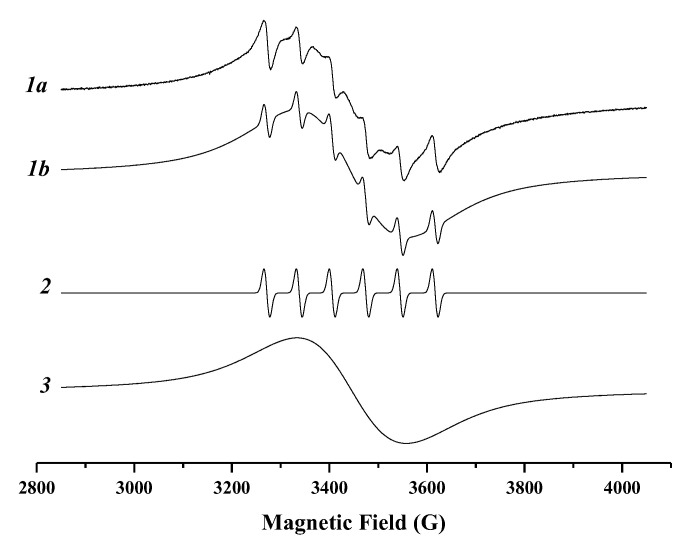
The EPR spectrum of Mn^2+^:ZnS:CdS/Zns nanoparticles measured at 293 K: 1a—the experimental spectrum, 1b—the sum of the 2 and 3 simulated spectra.

Figure 3 shows the EPR spectrum of Mn^2+^:Zn_0.46_Cd_0.54_S/ZnS QD-2 measured at 293 K. The experimental spectrum can be considered as a superposition of two signals. Spectral simulation of experimentally recorded ESR spectrum (spectrum 1a on Figure 3) made using Bruker SIMFONIA and WINEPR software package has allowed us to identify the Mn-related features in the EPR spectrum.

Signal I (spectrum 2 on Figure 3) consists of six well-resolved lines originated from the interaction between the unpaired electronic spin of Mn and the nuclear spin (I = 5/2). The hyperfine coupling constant A_iso_ = 68.8 ± 0.5 Gauss and g_iso_ = 2.0027 ± 0.0005 obtained from the simulation correspond to isolated Mn^2+^ ions located in the tetrahedral crystal field environment inside the core of the nanoparticle [36,37,38,39,40,41,42]. Thus, the signal I can be assigned to isolated Mn^2+^ ions presented inside the Zn_0.46_Cd_0.54_S core of QD-2. It should be noted that the values of magnetic resonance parameters of Mn^2+^ ions located in ZnS and CdS nanoparticles are very close [36,37,38,39,40,41,42]. So, it is very complicated to separate Mn^2+^ ions present in Zn-rich domains from Zn_x_Cd_1−x_-rich domains. The slight broadening of lines (linewidth ~ 13.0 Gauss) could be caused by Mn^2+^–Mn^2+^ dipolar interaction inside the Zn_0.46_Cd_0.54_S core.

Signal II (spectrum 3 on Figure 3) is a single broad background line. Such a broad spectral feature was observed in many Mn-doped nanoparticles and, usually, was assigned to Mn^2+^–Mn^2+^ strong dipolar interaction and/or exchange interaction [36,37,38,43,44]. It is reasonable to assume that the formation of ZnS shell leads to a displacement of some part of Mn^2+^ ions at the ZnS:CdS/ZnS core/shell interface, bringing them closer together. The proximity of Mn^2+^ ions at the interface causes Mn^2+^–Mn^2+^ strong dipolar and exchange interactions and, as a result, the single broad line is detected in EPR spectrum.

It is necessary to notice that another reason for the broadening of the ESR signal of Mn^2+^ ions could be crystal field distribution [37,45,46]. This suggests a distribution of hyperfine interaction, which could originate from isolated Mn^2+^ ions localized inside the nanocrystals, but near the surface in a strongly distorted crystal field.

Thus, according to the EPR results, the Mn^2+^ ions that could contribute to the PL band are the following: isolated Mn^2+^ ions inside the core of nanoparticle, dipolar interacting Mn^2+^ ions inside the core, Mn^2+^–Mn^2+^ strong dipolar interacting ions at the ZnS:CdS/ZnS core/shell interface, Mn^2+^–Mn^2+^ exchange interacting ions at the core/shell interface, dipolar interacting Mn^2+^ ions inside the core and core/shell interface, and Mn^2+^ ions in strongly distorted crystal field. Qualitatively, this is in accord with the observation of an inhomogeneous Mn^2+^ PL band due to the different ion localization in the host.

### 3.2. Femtosecond Transient Absorption (TA) Spectra

**Figure 4:**
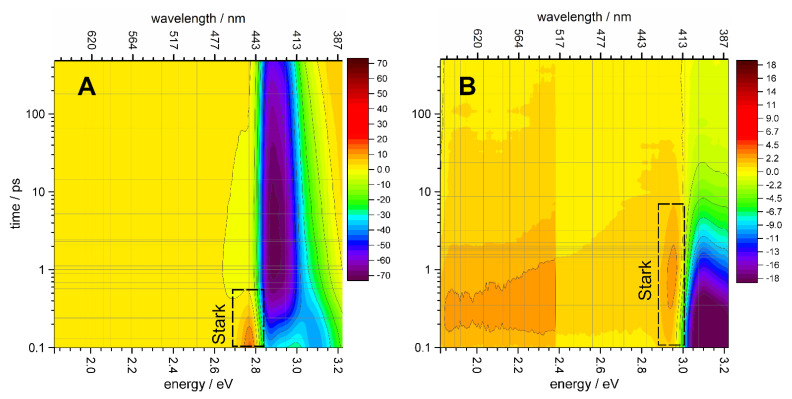
TA spectra of QD-1 (**A**) and QD-2 (**B**) samples in the form of the color map. TA signal in the range of ~1.95–2.35 eV (vertical black solid line is the border) is shown on the expanded scale with factor 3. ESA peaks corresponding to the Stark shift are indicated by dashed-line boxes.

The femtosecond pump-probe TA spectra of QD-1 and QD-2 samples for several representative time-delays are shown in Figure S3. The whole TA spectral matrices of the QD-1 and QD-2 samples are presented in Figure 4 in the form of color maps. The TA color maps show significant differences between the QD-1 and QD-2 samples. Firstly, a time-delayed absorption peak, marked in Figure 4B as “Stark” was detected in manganese-doped QD-2 sample at the red side of the exciton bleaching (BL) band, whereas no similar time-delayed peaks were seen in the undoped QD-1 sample (Figure 4A). In the undoped QD-1 sample, the peak of the Stark shift was recorded at the initial but not delayed time due to the biexciton interaction of the upper excitons with the band-edge exciton [47,48]. The decay of the Stark peak in QD-1 occured in parallel with the relaxation of the upper excitons (Figure 4A). Secondly, in the QD-2 sample with Mn^2+^ ions, the BL band associated with the band-edge exciton decayed much faster than the analogous band in the undoped QD-1 sample. This is qualitatively consistent with previously published femtosecond spectroscopy data for other quantum dots doped with Mn^2+^ [22,23,24,49] and suggests the exciton quenching by manganese. In the Mn^2+^-doped QD-2 sample, the development of the Stark peak took place concomitantly with the decay of the BL band of the band-edge exciton when the upper exciton states had already relaxed (Figure 4B). Since the Stark peak is associated with an electric field, its delayed appearance suggests a delayed growth of the electric field in the QD-2 sample, which may indicate oxidation of the Mn^2+^ ions by holes or reduction of Mn^2+^ by 1Se exciton electron. The intensity of the BL band is substantially controlled by the filling of the 1S_e_ electronic level [50]; therefore, the correlated decay of the BL band and the development of the Stark peak suggest that the 1S_e_ electron was captured by Mn^2+^. This observation means that a reduction of Mn^2+^ by 1Se electron could be preferable to the Mn^2+^ oxidation by the hole to Mn^3+^ at the stage of Stark peak development.

**Figure 5:**
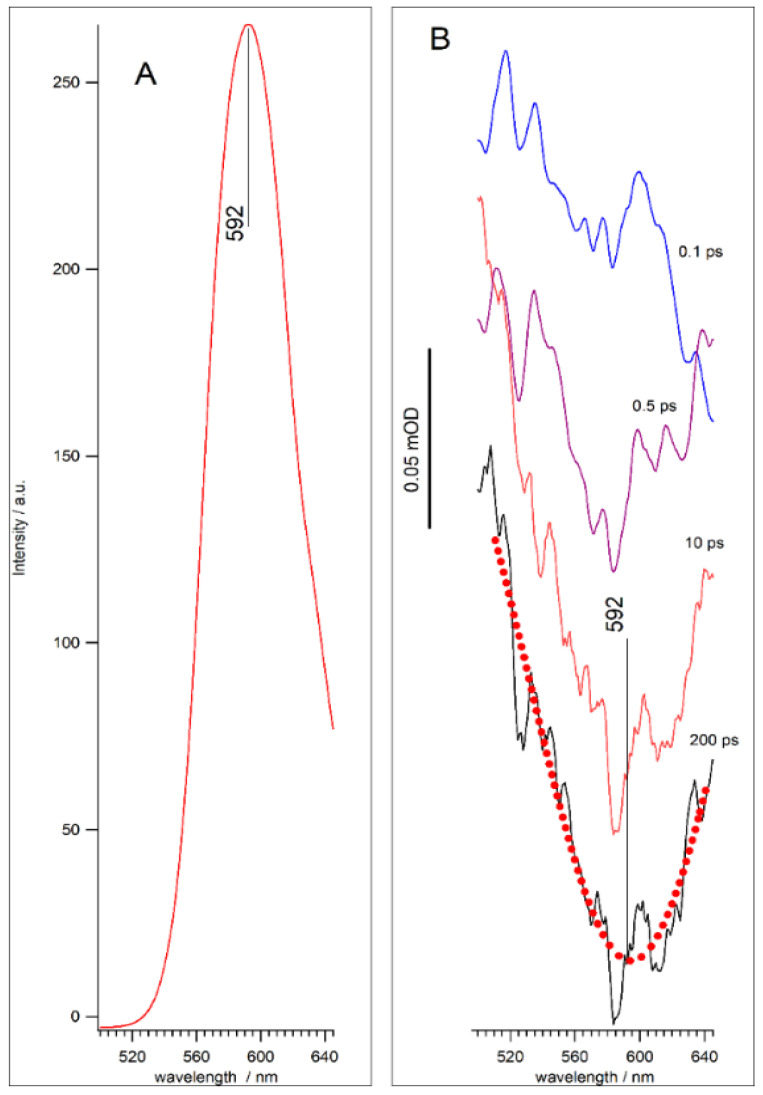
Mn^2+^ PL band (**A**) and transient spectra of QD-2 sample at different delay times (**B**). Red dotted line is Gaussian fitting curve.

Figure 5 shows the TA spectra of the QD-2 sample at different delays in the spectral domain around to Mn^2+^(^4^T_1_-^6^A_1_) transition. The negative peaks close to 590 nm can be tentatively attributed to the stimulated emission (SE) bands of Mn^2+^(^4^T_1_-^6^A_1_) (Figure 5B). The positions of the SE(592) band and the Mn^2+^(^4^T_1_-^6^A_1_) photoluminescence band coincided, as shown in Figure 5A. This coincidence suggests that the SE(592) band is associated with the luminescence of manganese. The wide positive background for the SE band can be attributed to the excited state absorption of charge carries in traps [51]. The SE(592) band in Figure 5B was detected at the sensitivity limit. The weak intensity of the SE(592) band is due to the small value of the transient dipole moment of the ^4^T_1_-^6^A_1_ transition. Weak SE(592) signal makes it difficult to quantitatively analyze the kinetics of manganese luminescence development. For this reason, in this work, we carried out a detailed analysis of the TA spectra in a wide spectral range for QDs doped and not doped with manganese.

### 3.3. Distribution of Relaxation Processes in the Photoinduced Transient Absorption

**Figure 6:**
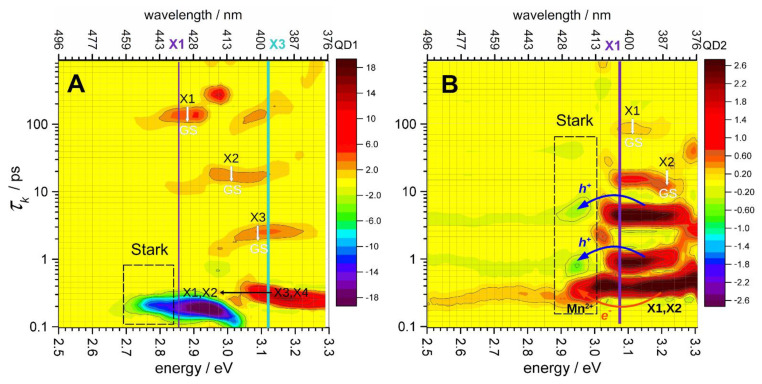
Distribution of the kinetic exponential components calculated by inverse Laplace transform of the TA dynamics of QD-1 (**A**) and QD-2 (**B**) in the range of 2.5–3.3 eV using the CONTIN program [52]. The amplitudes of the kinetic components are shown as colored bars. The Stark shift regions are indicated by dashed-line boxes, and the *X*_1_ and *X*_3_ peaks are marked by vertical lines. The red arrow in Figure 6B refers to the presumable process of Mn^2+^ → Mn^1+^ reduction, whereas the blue arrows mark the processes of Mn^1+^ oxidation and formation of the excited [Mn^2+^]^*^ in the 2 inhomogeneous Mn^2+^ locations.

The characteristic times of the observed relaxation processes in the TA dynamics were calculated using the program CONTIN [52]. This program implements the inverse Laplace transform to deconvolute non-monotonous relaxation into a distribution of exponential components

(1) $$\Delta A_{\nu }\left(t\right)=\underset{k}{\sum }a_{\nu ,k}\cdot e^{-t/\tau_{k}}$$

resulting in a quasi-continuous spectrum with the local smoothness determined by the Tikhonov–Phillips regularization. In contrast to a global fitting analysis, the CONTIN program determines the characteristic times of the observed relaxation processes independently at each probing frequency *ν* and can in principle determine how many processes are observed at different probing frequencies [53]. This is important for the analysis of coherent hot multi-exciton relaxation, which may proceed by several parallel channels. The results of CONTIN analysis are presented in Figure 6 in the form of spectrograms displaying the *a_ν,k_* distribution in the energy range between 2.5 and 3.3 eV. The absorption increase or decrease (negative or positive *a_ν,k_* in Equation (1)) is shown in red or blue in Figure 6, respectively.

### 3.4. Decomposition of Transient Absorption into Gaussian Components

According to Norris and Bawendi, among four Gaussian components distinguishable in the linear absorption spectra (Figure 1, Table 1), the lowest *X*_1_ can be attributed to the 1S_e_-1S_3/2_ transition, whereas other three represent combinations of several transitions: the second *X*_2_ includes 1S_e_-2S_3/2_ and 1S_e_-1S_1/2_, the third *X*_3_ includes 1P_e_-1P_3/2_ and 1S_e_-2S_1/2_, and the broad fourth *X*_4_ may include a combination of 1P_e_-1P_1/2_, 1P_e_-1P_5/2_, and 1S_e_-3S_1/2_ transitions [54]. Interpretation of the TA spectra in terms of the spherical electron and hole envelope wave functions is complicated by the overlap of bleach and induced absorption features. Several other effects have to be taken into consideration: the fine-structure splitting of the band-edge transitions, the multi-particle inter-exciton interactions, the statistical distribution of multi-exciton states, and the complicated Stokes shift structure [55,56,57]. The simplest quantitative interpretation is the Stark redshift of the lowest *X*_1_ peak (1S_e_-1S_3/2_ transition), the biexciton interactions of which were previously thoroughly analyzed [58]. The observed biexciton red shift Δ_XX_ of the *X*_1_ peak in the TA spectra of CdSe nanoparticles of various sizes upon pumping into the 1P exciton (the *X*_3_ peak) was in the range of 9–18 meV, whereas the redshift was almost zero upon pumping into the 1S band-edge exciton (the *X*_1_ peak) [56,57]. Zhang et al. [50] analyzed the absorption changes in pump–probe measurements by using double-sided Feynman diagrams techniques and suggested an approximation where the TA spectra are modeled in the visible range by a sum of three Gaussian functions

(2) $$\begin{array}{r} \Delta A(\omega )\propto -\sum_{k=1,2,3}\alpha_{k}G_{k}(\omega -\omega_{k})+\alpha_{1}\left[G_{1}(\omega -\omega_{1}+\Delta_{1})-G_{1}(\omega -\omega_{1})\right]\\ +0.5\alpha_{2}G_{2}(\omega -\omega_{2}+\Delta_{2})+\alpha_{3}G_{3}(\omega -\omega_{3}+\Delta_{3})\; \end{array}$$

where *G*(*ω* − *ω_k_*) is the Gaussian function

$$G_{k}\left(\omega -\omega_{k}\right)=exp\left(-{\left(\omega -\omega_{k}\right)}^{2}/2w_{k}^{2}\right)$$

The peak positions *ω_k_* and the widths *w_k_* of the unshifted Gaussian functions $G_{k}\left(\omega -\omega_{k}\right)$ are usually taken from the decomposition of the *X*_1_, *X*_2,_ and *X*_3_ bands in the linear absorption spectrum. Because the *X*_2_ and *X*_3_ bands comprise combinations of several transitions and their bleach and induced absorption features essentially overlap in the TA spectra, we simplified Equation (2), confining ourselves to the linear term of the expansion of *G*_2_(*ω* − *ω*_2_ + Δ_2_) and *G*_3_(*ω* − *ω*_3_ + Δ_3_) in the Taylor series:

(3) $$\begin{array}{ll} \Delta A(t,\omega ) & =\sum_{k=1,2,3}B_{k}(t)\cdot G_{k}(\omega -\omega_{k})+\\ & C_{1}(t)\cdot \left[G_{1}(\omega -\omega_{1}+\delta_{1})-G(\omega -\omega_{k})\right]+\sum_{k=2,3}C_{k}(t)\cdot G_{k}^{\prime }(\omega -\omega_{k}) \end{array}$$

**Figure 7:**
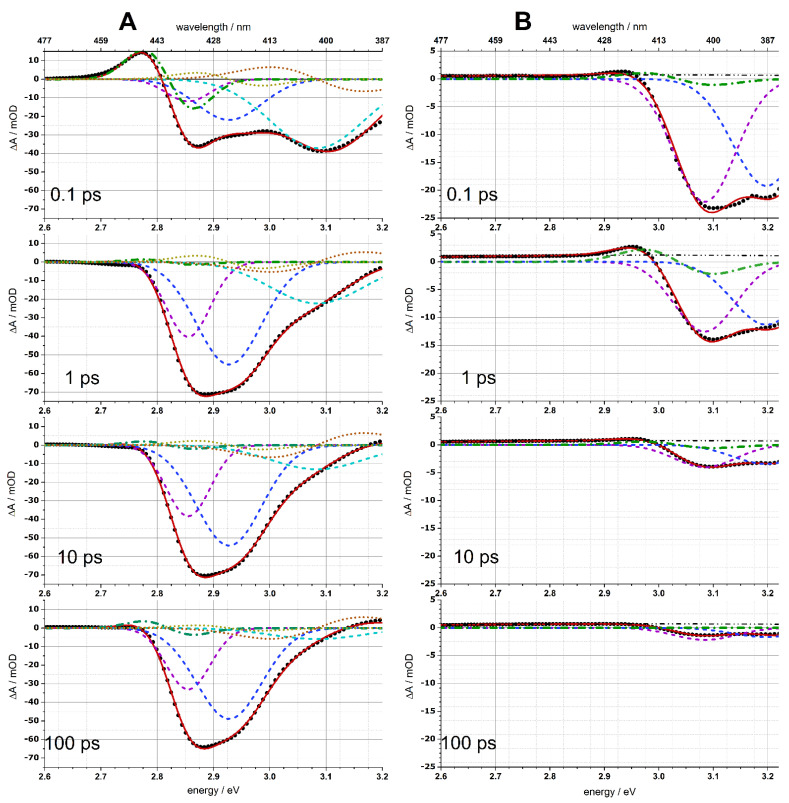
TA spectra at different delay times after 30 fs, 360 nm pump. (**A**) QD-1, without Mn^2+^ ions. (**B**) Mn^2+^-doped QD-2. TA experimental data are shown as black circle marks. Solid red lines represent the fitting model (Equation (3)) with parameters in Table 1. BL bands are *X*_1_ (violet, dashed), *X*_2_ (blue, dashed), *X*_3_ (cyan, dashed). Stark spectral feature of the band-edge exciton (*X*_1_) is indicated by S-like dash-dotted green lines. The Stark features of *X*_2_ and *X*_3_ excitons are indicated by yellow and brown dots, respectively.

**Figure 8:**
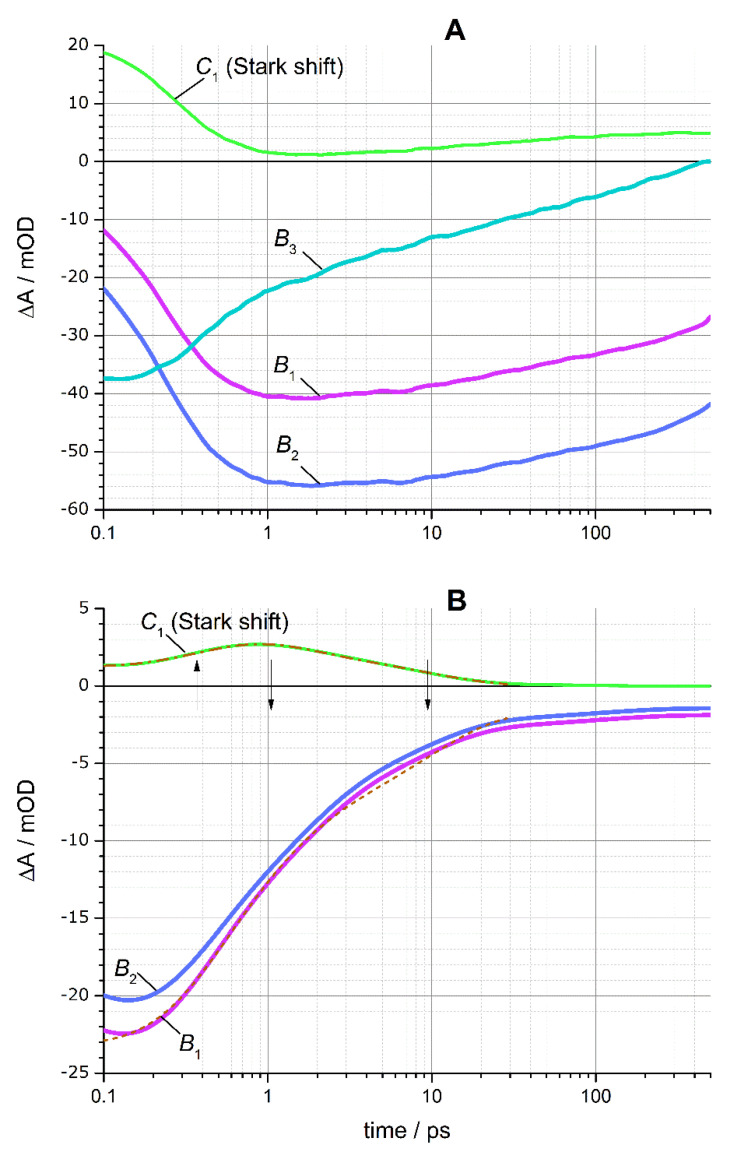
Kinetics of the *B*_1,2,3_(*t*) bleach peaks amplitude and the *C*_1_(*t*) amplitude associated with the electrochromic Stark shift of the band-edge exciton *X*_1_. (**A**) QD-1 sample without Mn^2+^ ions. Green solid line is the experimental Stark shift. (**B**) QD-2 sample doped with Mn^2+^. Green solid line is the experimental Stark shift, red dash-dotted line is the model (5). The fitting of TA spectra from Figure 5A,B by 3 and 2 Gaussian components, respectively, was done using Equation (3) as illustrated in Figure 7. The kinetic model (4) of sequential electron-hole transfer in the Mn^2+^-doped QD-2 sample is shown by dashed lines according to Equation (5). Up and down arrows indicate the characteristic times of electron and hole transfer processes, respectively.

Here *G′_k_*(*ω* − *ω_k_*) are the first derivatives of *G_k_*(*ω* − *ω_k_*), which correspond to the spectral features of the Stark shifts of *X*_2_ and *X*_3_; the positions of the peaks *ω_k_* and the widths *w_k_* of the Gaussian functions *G_k_* were determined from the decomposition of the linear absorption spectra into Gaussians (see Figure 1 and Table 1, Figure S4); the amplitudes *B_k_*(*t*) and *C_k_*(*t*), corresponding to bleaching and Stark shift features, respectively, were found using linear least-squares regression of the TA spectra in the entire time range shown on the TA colormaps in Figure 5. The magnitude of the electrochromic shift δ_1_ of the band-edge exciton was found as a single parameter for the entire matrix of TA spectral changes by nonlinear minimization, as described previously [31,53]. Figure 7 shows the fitting of the TA spectra by Equation (3) for several selected delay times. The amplitudes of bleach peaks *B*_1_(*t*), *B*_2_(*t*), and *B*_3_(*t*) can be assigned to the populations of the three predominant exciton states 1S_e_ − 1S_3/2_, 1S_e_ − 2S_3/2_, and 1P_e_ − 1P_3/2_, respectively [54]. The dynamics of the *B*_1,2,3_(*t*) bleach peaks amplitude and the *C*_1_(*t*) amplitude associated with the electrochromic Stark shift of the band-edge exciton obtained as a result of the simulation are shown in Figure 8.

Figure 8A shows that in the QD-1 sample, the growth of the *X*_1_ and *X*_2_ bleach amplitudes *B*_1_(*t*) and *B*_2_(*t*) in the time range of ≤0.5 ps occurred in parallel with a decay of the Stark shift *C*_1_(*t*) of the band-edge exciton *X*_1_. Since the *X*_3_ bleach amplitude *B*_3_(*t*) did not change significantly in the time range up to 0.5 ps, the Stark shift *C*_1_(*t*) is attributed mainly to the *X*_4_-*X*_1_ biexciton interaction; such attribution is consistent with a large magnitude of the Stark shift (δ_1_ = 70 meV, see Table 1), which substantially exceeds the estimates of 10–20 meV obtained for the *X*_3_-*X*_1_ biexciton interaction [56,57,58,59]. In the QD-1, the decrease of the Stark shift to almost zero in parallel with the relaxation of the upper excitons indicates that the terminal stage of electronic transitions *X*_4_ → *X*_3_ → *X*_2_, *X*_1_ proceeds at a timescale of ~300 fs in agreement with previous studies of hot exciton relaxation in different QDs [60,61,62]. The slow decrease of the *B*_3_(*t*) amplitude in the time range of 1–100 ps proceeded in parallel with a small increase of the Stark shift *C*_1_(*t*); both effects can be explained by the trapping of free charges on the surface of the nanoparticle [31,56].

Figure 8B shows that in the QD-2 sample with Mn^2+^ ions, a fast decay of the X_1_, X_2_ bleach peaks took place in the time window of ≤1 ps, and in the same time scale a substantial Stark shift of the X_1_ band was developing. In this sample, the X_3_ bleaching was not resolved; however, based on the increase in the X_1_/X_2_ bleaching at the shortest delays of ≤200 fs, the presence of a small contribution of the X_3_ → X_2_/X_1_ transition, *ξ*_3_, is suggested for this sample.

The transient dynamics of the QD-2 sample differed significantly from that of the QD-1 in two aspects. First, the *X*_1_ bleaching disappeared in the QD-2 sample at the timescale of 1 ps, whereas the *X*_1_ exciton in the QD-1 sample decayed three orders of magnitude slower in the nanosecond time scale. Second, the electrochromic shift of the *X*_1_ band *C*_1_(*t*) in QD-2 was small at the shortest delays (because of the small yield of the *X*_4_ exciton in this sample), but *C*_1_(*t*) increased at delays of ~1 ps. The decay of the *X*_1_/*X*_2_ BL amplitude is somewhat ahead of the kinetics of the Stark shift disappearance. The concomitant decay of the *X*_1_/*X*_2_ bleach and the transient rising of the Stark shift suggests that the energy transfer from the *X*_1_ and *X*_2_ excitons to the Mn^2+^ ions proceeds a two-step charge transfer mechanism, as was suggested previously by Gahlot et al. [28]. We attributed the kinetics of the transient Stark shift upturn to transiently recharging Mn^2+^ ions, the localized charges of which induced a large electrochromic band shift of the *X*_1_ band (*ħδ*_1_ = 0.1 eV, see Table 1) due to strong charge-exciton interaction.

**Scheme 1:**
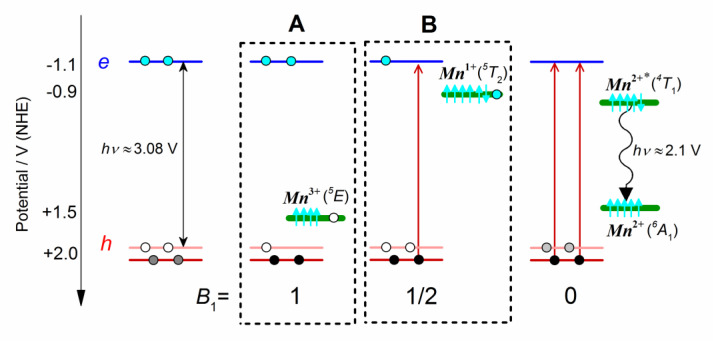
Electronic configurations of the 2 alternative charge-transfer intermediates (**A**,**B**) that can be responsible for a 2-step energy transfer from the *X*_1_ exciton to Mn^2+^ ions ^+^ and the overall ^6^A_1_ → ^4^T_1_ transition. The electronic states of Mn^3+^ and Mn^+^ ions correspond to the ^2^*E* (3*d*^4^) and ^5^*T*_2_ (3*d*^6^) configurations, in accordance with the DFT calculations [69].

The sequential transfer of two charges may occur in two different ways: (A) the Mn^2+^ ion is oxidized to Mn^3+^ by a hole transfer from the valence band, after which an electron is transferred to the Mn^3+^ from the conduction band; (B) alternatively, the Mn^2+^ ion is reduced in the first step to Mn^1+^ by an electron transfer from the conduction band, after which a hole is transferred from the valence band (see boxes A and B in Scheme 1). The first mechanism prevails if the redox potential of the Mn^3+^/Mn^2+^ transition lies below the redox potential of the valence band, and the second case may take place if the redox potential of the Mn^2+^/Mn^1+^ transition lies above the redox potential of the conduction band.

The redox potentials of the conduction band were determined for the CdS and ZnS nanoparticles by direct electrochemical measurements in the range between −2.15 and −2.3 V vs NHE and were found to be weakly dependent on the size of nanoparticles, whereas the redox potentials of the valence band were in the range from +0.55 to +0.75 V vs NHE [19]. The reduction potentials for the Mn^2+^/Mn^+^ transition in several organic complexes varied between−0.7 and−1.3 V vs. NHE [63,64,65,66], and the reduction potentials for the Mn^3+^/Mn^2+^ couple in an aqueous solutions of various ionic ligands were between +0.8 and +1.5 V vs. NHE [67]. This means that the energy level of the Mn^2+^/Mn^+^ transition is most likely within the bandgap of the QD-2 particle, while the Mn^3+^/Mn^2+^ level is submerged in the valence band. Beaulac and Gamelin calculated energy levels of the Mn^2+^ orbitals for different Mn^2+^-doped semiconductors and found out that the energy level of 3*d*^5^ orbital (Mn^2+/3+^ transition) is located deep in the valence band, whereas the unoccupied Mn^2+^ orbitals participate in exchange interactions with electrons of the conduction band, allowing Mn^2+^ to be a 3*d*-based electron acceptor (Mn^2+/+^ transition) [68].

Kinetic modeling of the absorption dynamics gives allows us to distinguish which of the two probable mechanisms the reaction follows. The B_1_ amplitude of the *X*_1_ band is determined by the filling of the 1S_e_ electron and 1S_3/2_ hole states. The scavenge of holes (mechanism A) has little effect on the BL amplitude [47], the relative extent of which is indicated in Scheme 1 by the dimensionless coefficient *θ* = 1. Assuming that all 1S_e_ states are initially filled, the loss of one electron from the *X*_1_ band in the result of the Mn^2+^ reduction (mechanism B) leads to a two-fold decrease in the BL amplitude due to a decrease in the population of the 1S_e_ electronic state (*θ* = 1/2). To simplify the kinetic model, we assume that the *X*_1_ and *X*_2_ excitons are in thermal equilibrium and the transfer of the second electron to Mn^2+^ is compensated by the electron exchange between the 1S_e_ and 2S_e_ states. The kinetic scheme (4) includes therefore three transitions

(4) $$X_{3}\overset{\;k_{3}\;}{\to }X_{1}\overset{\;m_{1}\;}{\to }X_{\mathrm{CT}}\overset{\;m_{2}\;}{\to }X_{E}$$

where *X*_3_ and *X*_1_ are the generalized populations of the *X*_3_ and *X*_2_/*X*_1_ excitons, *X*_CT_ is the population of the charge transfer intermediate A or B in Scheme 1, and *X*_E_ is the population of the final ^4^*T*_1_ excited state of Mn^2+^. The transitions between these states are described by the kinetic constants *k*_3_, *m*_1_, and *m*_2_ for the first order reactions. The kinetics of the *B*_1_(*t*) bleach amplitude and the *C*_1_(*t*) amplitude of the *X*_1_ shift are

(5) $$\begin{array}{l} B_{1}(t)=a_{1}\left(X_{1}+\theta \cdot X_{\mathrm{CT}}\right)\\ C_{1}(t)=c_{3}X_{3}+c_{0}X_{\mathrm{CT}} \end{array}$$

Here *a*_1_ is the absorbance of the *X*_1_ bleach peak, *c*_3_ and *c*_0_ are the electrochromic shifts of the *X*_1_ band due to the *X*_3_-*X*_1_ biexciton and the charge–exciton interactions, and *θ* is the dimensionless coefficient, the value of which is determined by the reaction mechanism. Numerical analysis showed that the kinetic model should take into account the heterogeneity of the sample, the presence of which also follows from the EPR data (Figure 3) and the CONTIN kinetic analysis (Figure 6). For this purpose, the model considered three fractions of the Mn^2+^ locations. The first two fractions, *f*_A_, and *f*_B_ (Table 2), differed in the magnitude of the *m*_2_ charge transfer constant (two down arrows in Figure 8B), and in the third fraction *f*_C_ there was no manganese in the nanoparticle (*m*_2_ = 0).

The found value of coefficient *θ* = 0.49 (Table 2) agrees with the electron-to-hole mechanism of energy with the intermediate Mn^2+^ reduction (variant B in Scheme 1). The model includes the fast *X*_3_ → *X*_1_ transition (*k*_3_ = 9.5 ps^−1^) in 19% of the particles, the slower Mn^2+^ reduction (*m*_1_ = 2.7 ps^−1^), and the formation of excited [Mn^2+^]* state due to the slowest electron transfer reaction (*m*_2A_ = 0.96 ps^−1^ in the fraction *f*_A_ = 0.39 and *m*_2B_ = 0.1 ps^−1^ in the fraction *f*_B_ = 0.55). The model kinetic curves are shown in Figure 8B by dashed curves.

Different time-resolved measurements of the Mn^2+^ excitation time ranging from a few hundred fs to a few ns were reported in the literature. Chen et al. [23] estimated the time constant τET of ~60 ps for the exciton to Mn^2+^ energy transfer in CdS/ZnS QDs employing the pump–probe transient absorption technique to measure the exciton relaxation dynamics in doped and undoped QDs. A near-IR probe wavelength primarily monitoring the intraband transition of excitons was used [23]. S. Taguchi et al. [70] registered the τET value of ~2 ns in Mn^2+^-doped core/shell CdS/ZnS QDs. TA bleaching of the band-edge exciton was measured in doped and undoped QDs, and a model including the bimolecular recombination of e–h pairs and three-particle Auger recombination was used to extract τET value [70]. Chung et al. [71] measured a picosecond TA and subnanosecond fluorescence dynamics in Mn-doped ZnS nanoparticles. They reported the energy-transfer process to Mn^2+^ impurities occurring on the time scale of 700 ps [71]. Olano et al. studied energy-transfer dynamics in Mn^2+^-doped ZnSe QDs by registering the photoluminescence using time-integrated, time-resolved spectroscopic techniques and femtosecond TA spectroscopy. Mn^2+^ doping substantially shortens the average lifetimes of the band-edge excitonic state as well as shallow trap states, which suggests the energy transfer from ZnSe to Mn^2+^ follows two mechanisms, one through trap states and another without, on the time scale of tens of ps [72]. Shibata et al. analyzed the radiative and non-radiative recombination processes in the Mn-doped CdSe QDs monitoring luminescence by streak camera technique. They found out that the exciton energy transfer to Mn^2+^ 3d^5^ electrons occurs on a time scale of 20 ps [73]. Hsiang-Yun Chen et al. [74] studied dynamics of energy transfer in Mn-doped CdS/ZnS core/shell QDs via transient absorption measurement of exciton relaxation dynamics. They found the strong dependence of the Mn^2+^ excitation rate on doping location. It was found that the band-edge exciton decay of Mn^2+^(3d^5^) occurs as a multiexponential process. The average quenching time of the Mn^2+^ exciton is in the range 3.8–80 ps, depending on the concentration of Mn^2+^ ions and their location in the QD matrix. The faster time constant can be ~0.56 ps with the relative amplitude of ~62% [74]. The cited works [23,70,71,72,73,74] suggest that Mn^2+^ excitation can occur due to the non-radiative Auger-like exciton recombination, Dexter, and Förster energy-transfer mechanism [10,11,12,15,19,21,23,24,25,26,65,66,67,68,69] from an exciton to the d-d levels of manganese. An alternative way to excite Mn^2+^ may be a charge transfer mechanism [49]. K. Gahlot et al. [28] suggested that oxidation of Mn^2+^ occurs with a characteristic time of τ_p_~100–200 fs for Mn^2+^:Cd_x_Zn_1−x_Se QDs. Our observation of redox reaction with a characteristic time of 1/m_1_ ~ 380 fs is in accord with τ_p_ ~ 200 fs. In contrast to the assumption about the oxidation of Mn^2+^ by the hole at the first step of the redox process made in [28], the results of this work suggest the reduction of Mn^2+^ is the first event in the manganese excitation process. According to K. Gahlot et al. [28], excited Mn^2+^* is formed from the intermediate charge transfer state of Mn^3+^ with a characteristic time of 300–800 ps [28]. This time value of 300–800 ps significantly exceeds the characteristic time of excitation of Mn^2+^ obtained in this work, which is approximately 1ps. The observation of stimulated emission of manganese ions SE(592) for Mn^2+^:Cd_0.5_Zn_0.5_S in the time window of 1 ps in the present work, and recently, [26] suggested that the excitation of manganese occurs much faster than it is assumed in [28] for Mn^2+^:Cd_x_Zn_1−x_Se QDs. Kinetics of the Stark peak at the time scale of ~1 ps and the observation of SE(592) at short times suggest the process of manganese excitation is completed by oxidation of Mn^1+^ by a hole (Scheme 1). The assumption about the reduction of Mn^2+^ to Mn^+1^ is consistent with the observation that manganese-doped QDs in QD solar cells has shown an enhancement in energy conversion efficiency by 20% compared to unalloyed analog [49,75].

## 4. Conclusions

The femtosecond transient absorption (TA) spectra of quantum dots (QDs) of the manganese-doped Mn^2+^:Zn_~0.5_Cd_~0.5_S/ZnS alloy reveals a specific feature that manifests itself as an absorption peak that appears with a time delay. The delayed absorption peak has spectral features of the electrochromic Stark shift of the band-edge exciton. The delayed development and decay kinetics of this Stark peak in manganese-doped QDs significantly distinguish it from the known Stark peak associated with electrochromic shift caused by exciton–exciton interactions in undoped QDs at the initial time delays. The delayed Stark shift in QDs doped with Mn^2+^ suggests the development of the electric field in QDs due to charge transfer. Charge transfer processes can be attributed to the reduction of Mn^2+^ by electrons from the 1S_e_ state, followed by the oxidation of Mn^1+^ by holes with the formation of excited Mn^2+^(d^5^)* ions. The charge transfer mechanism solves the problem of a significant mismatch between the energy gaps of the band-edge exciton and the excited manganese and may not contradict the Wigner rule. This mechanism can provide a high rate of manganese excitation, which manifests itself in the TA spectra at short delay times. The revealed Stark peak suggests a charge-transfer mechanism of Mn^2+^ excitation by the QDs band-edge exciton in contrast to the non-radiative energy-transfer mechanism, which does not imply the development of an electric field in the QDs.

## Figures and Tables

**Table 1:** Gaussian decomposition of the absorption and TA spectra of QD-1 and QD-2 samples.

	*X*_1_			*X*_2_		*X*_3_		*X*_4_	
	*ħω*_1_ eV	*ħw*_1_ eV	*ħδ*_1_ eV	*ħω*′_1_ eV	*ħw*′_1_ eV	*ħω*_2_ eV	*ħw*_2_ eV	*ħω*_3_ eV	*ħω*_3_ eV
QD-1 sample	2.85	0.03	0.08	2.92	0.05	3.08	0.13	3.45	0.20
QD-2 sample	3.08	0.05	0.10	3.20	0.06	3.44	0.17	4.27	0.36

**Table 2:** Kinetic model of the *B*_1_ bleach peak and the Stark shift *C*_1_ changes of the QD-2 sample.

Fractions				Kinetic Constants				
*f*_A_	*f*_B_	*f*_C_	*ξ*_3_	1/*k*_3_ (ps)	1/*m*_1_ (ps)	1/*m*_2A_ (ps)	1/*m*_2B_ (ps)	*θ*
0.39	0.55	0.06	0.19	0.11 ± 0.04	0.38 ± 0.02	1.03 ± 0.1	9.6 ± 0.9	0.49

## Supplementary Materials

The following are available online at https://www.mdpi.com/article/10.3390/nano11113007/s1, Figure S1: TEM images of QD-1 and QD-2 samples; Figure S2: Absorption, PLE and PL spectra of QD-2; Figure S3: Femtosecond Pump-Probe TA spectra of QD-1 and QD-2 samples; Figure S4: Decomposition of TA spectra into Gaussian peaks in the approximation of the model of exciton and biexciton transitions.
