# Supplementary material for: Ultrafast Quenching of Excitons in the ZnxCd1−xS/ZnS Quantum Dots Doped with Mn2+ through Charge Transfer Intermediates Results in Manganese Luminescence
Source: Nanomaterials (Basel). 2021 Nov 9;11(11):3007. doi: 10.3390/nano11113007 (PMC8618633; doi:10.3390/nano11113007)
Supplement: Supplementary file 1 [file nanomaterials-11-03007-s001.zip › nanomaterials-1432867-supplementary.pdf]

Supplementary Information

Ultrafast Quenching of Excitons in the $Zn_xCd_{1-x}S$/ ZnS Quantum Dots Doped with Mn^{2+} through Charge Transfer Intermediates Results in Manganese Luminescence

Dmitry Cherepanov [1], Andrei Kostrov [1], Fedor Gostev [1], Ivan Shelaev [1], Mikhail Motyakin [1], Sergei Kochev [2], Yuriy Kabachii [2] and Victor Nadtochenko [1,3,*]

[1] N.N. Semenov Federal Research Center for Chemical Physics, RAS, Kosygin st. 4, Moscow 119991, Russia; tscherepanov@gmail.com (D.C.); andreikostrov@rambler.ru (A.K.); boatsween@yandex.ru (F.G.); shelaev@bk.ru (I.S.); motyakin@hotmail.com (M.M.)

[2] A.N. Nesmeyanov Institute of Organoelement Compounds, RAS, 28 Vavilov St., Moscow 119991, Russia; kochew@ineos.ac.ru (S.K.); kabachi@ineos.ac.ru (Y.K.)

[3] Department of Chemistry, Lomonosov Moscow State University, Leninskiye Gory 1-3, Moscow 119991, Russia

* Correspondence: nadtochenko@gmail.com; Tel.: +7-(499)-137-66-76

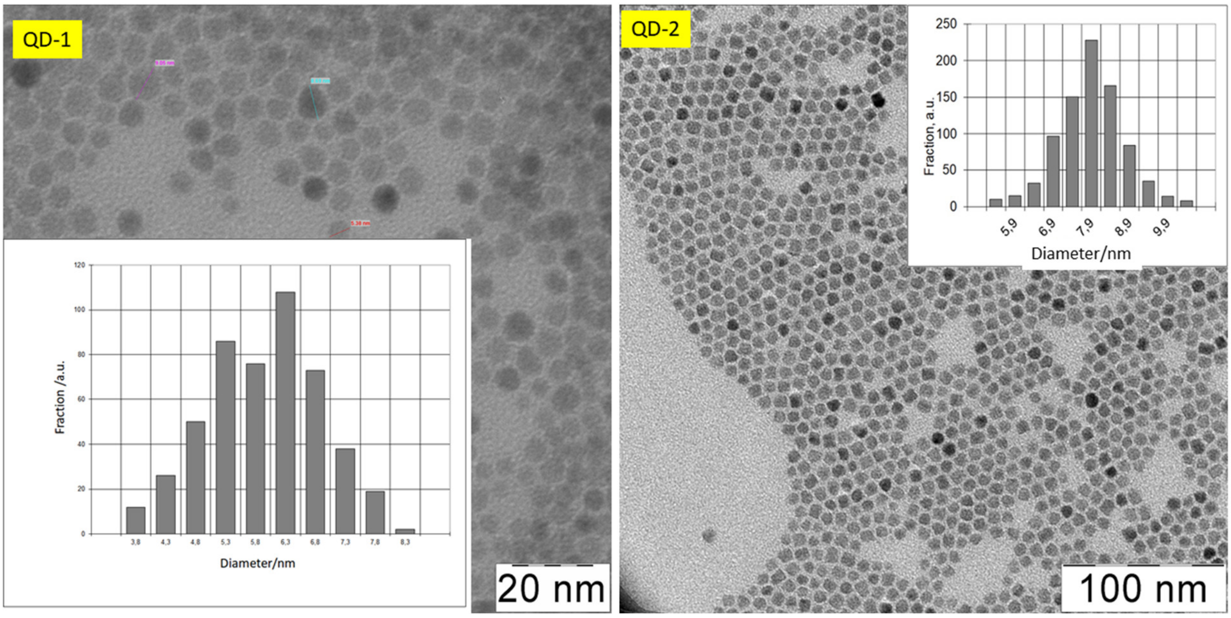

Figure S1. TEM images of QD-1 and QD-2 samples.

1. Chemicals

Zinc acetate dihydrate ($Zn(OAc)_2$•$2H_2O$, 98%), cyclohexane (99+%, for spectroscopy), 1-octadecene (ODE, 90%, tech.,), tetramethylammonium hydroxide (TMAH, 25 wt% in methanol), manganese(II) chloride ($MnCl_2$, anhydrous, 99%), CdO (99.5%), oleic acid (90%, tech.), oleylamine (OlAm, C18 content 80-90%) cadmium acetate dihydrate ($Cd(OAc)_2$•$2H_2O$, ≥98%), stearic acid (HSt, 95%) were purchased from Merck and used as received. Sulfur (reagent grade, 100 mesh) and magnesium turnings (Mg, 99%) were purchased from Sigma-Aldrich. Anhydrous methyl alcohol (MeOH) was prepared from chemically pure grade MeOH by boiling with magnesium methylate. Manganese stearate ($MnSt_2$) was synthesized from $MnCl_2$, TMAH and HSt in anhydrous MeOH. OlAm was degassed at 60 °C for 30 min under vacuum in Schlenk tube and stored under argon.

2. Installation for the synthesis of nanocrystals

Nanocrystal synthesis was carried out under argon atmosphere in a three-neck 100 or 50 mL round-bottom flask equipped with a magnetic stirrer, a reflux condenser and a rubber stopper with a thermocouple and a heating mantle connected to the thermostat. The open end of the reflux condenser is connected either to a vacuum line or to an argon gas pipe, equipped with an exit of argon into the atmosphere through a bubble counter, to prevent accidental entry of air into the flask during the experiment.

3. Synthesis of Zn and Cd precursor mixture

The Zn and Cd precursor mixture was prepared by the following typical procedure. A mixture of $Cd(OAc)_2$•$2H_2O$ (95.4 mg, 0.358 mmol), $Zn(OAc)_2$•$2H_2O$ (78.6 mg, 0.358 mmol), 0.523 mL of oleic acid (455 mg, 1.65 mmol) and 1 mL of ODE in the 25 mL Schlenk tube was stirred at 130 C for 30 min under argon flow, evacuated for 20 min, then cooled and mixed with 7.5 mL of ODE.

4. Synthesis of Zn precursors

The first Zn precursor was prepared by the typical procedure which includes slow heating of mixture of $Zn(OAc)_2$•$2H_2O$ (128.2 mg, 0.584 mmol), 0.380 mL of oleic acid (338 mg, 1.20 mmol) and ODE (0.5 mL) in the 25 mL Schlenk tube under vacuum, from ambient temperature up to 130 °C, kept under this temperature for 20 min, and cooling. Next, the mixture was mixed with 2 mL of ODE, degassed under vacuum at 100 °C, cooled down to ambient temperature and stored under argon.

The second Zn precursor was prepared from the mixture of $Zn(OAc)_2$•$2H_2O$ (335.4 mg 1.582 mmol), 1.034 mL of oleic acid (920 mg, 3.26 mmol) and 3 mL of ODE by the same procedure.

5. The typical procedure for sulfur stock solution preparation

The sulfur stock solution was prepared by dissolution of 48.7 mg (1.518 mmol) of sulfur in 8 mL of degassed ODE in the 25 mL Schlenk tube at 135°C for a 15-20 min. After cooling to room temperature, the solution was stored under argon atmosphere. The sulfur stock solution described here was used for the synthesis of Mn^{2+}-doped ZnCdS/ZnS core/shell QDs (QD-4).

6. Synthesis of $Zn_xCd_{1-x}S$ QDs (QD-1)

A mixture of Cd(OAc)2•2H2O 95.4 mg (0.358 mmol), Zn(OAc)2•2H2O 78.6 mg (0.358 mmol), 0.523 mL of oleic acid and 1 mL of ODE in the 50 mL Schlenk tube was stirred at 130 C for 30 min under argon flow, evacuated for 20 min, then cooled and mixed with 7.5 mL of ODE. Sulfur 23 mg (0.719 mmol) was dissolved in 1.8 mL of degassed ODE in the 25 mL Schlenk tube at 135°C for a 15-20 min. After that the solution was cooled to room temperature and stored under argon. Oleic acid 470 μL was mixed with ODE 0.52 mL, degassed under vacuum and kept in argon. The Zn and Cd precursor mixture was loaded into the 100 mL three-neck round-bottom flask. The mixture was withstanded under vacuum for 20 min while heating to 100°C. Then the flask was filled with argon. After that the flask was heated to 305°C, and OlAm 1 mL and the sulfur solution were injected successively by the syringe. The mixture was stirred at 310°C for 10 min. Next, the prepared oleic acid solution in ODE was added; the mixture was stirred for another 10 min and cooled to room temperature. Acetone was added to the mixture in a ratio of 1: 1.5. The resulting precipitate was separated from liquid by centrifugation at 5000 rpm. The liquid was discarded. The precipitate was redispersed in heptane and precipitated with acetone. The precipitate was separated from liquid by centrifugation, and the liquid was discarded. The procedure of re-dispersion-precipitation-separation was repeated three times. Final precipitate was dispersed in cyclohexane. Non dispersible residue was separated by centrifugation at 8000 rpm (~20.000g) for 5 min and discarded. As a result, 80 mg of the Sample QD-1 nanocrystals were obtaned as a colloid in cyclohexane.

7. Synthesis of Mn^{2+}-doped **$Zn_xCd_{1-x}S$/ZnS** QD-2.

2.5 ML of the Zn and Cd precursor mixture, 11.7 mg (0.019 mmol) of $MnSt_2$ and 7.5 mL of ODE were loaded into the 100 mL the three-neck round-bottom flask. The mixture was kept under vacuum for 20 min while heating to 100°C, and then the flask was filled with argon. After that the flask was heated up to 305°C, and 0.136 mL of OlAm and 1 mL of the sulfur stock solution was injected successively by the syringe; the mixture was stirred at 310°C for 20 min and cooled to 240°C. Then, without isolation, the formed nanocrystal cores were coated with a ZnS shell. OlAm 1.023 mL (828 mg, 3.097 mmol) was added to the mixture at that temperature, and then the remaining sulfur stock solution and the second Zn precursor heated to 100°C were injected alternately in five steps (0.55, 0.89, 1.32, 1.83 mL, and the rest). The period between each injection was 10 min. After the fourth one the reaction mixture was kept at this temperature for 10 min and finally was cooled down to the ambient temperature. Finally, 153 mg of nanocrystals QD-2 were yielded as dispersion in cyclohexane.

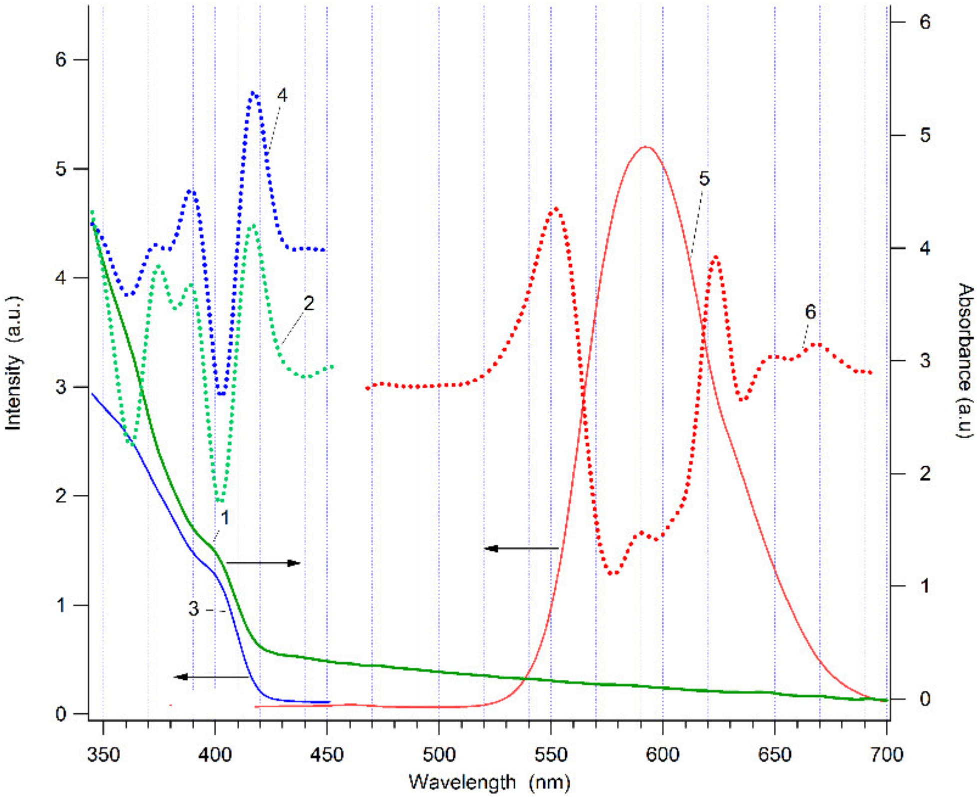

Figure S2. Absorption (1), PLE(3) and PL(5) spectra of QD-2.

Luminescence spectra were excited at 360 nm. Absorption (2), PLE(4) and PL(6) spectra of QD-2 in the form of the second derivative.

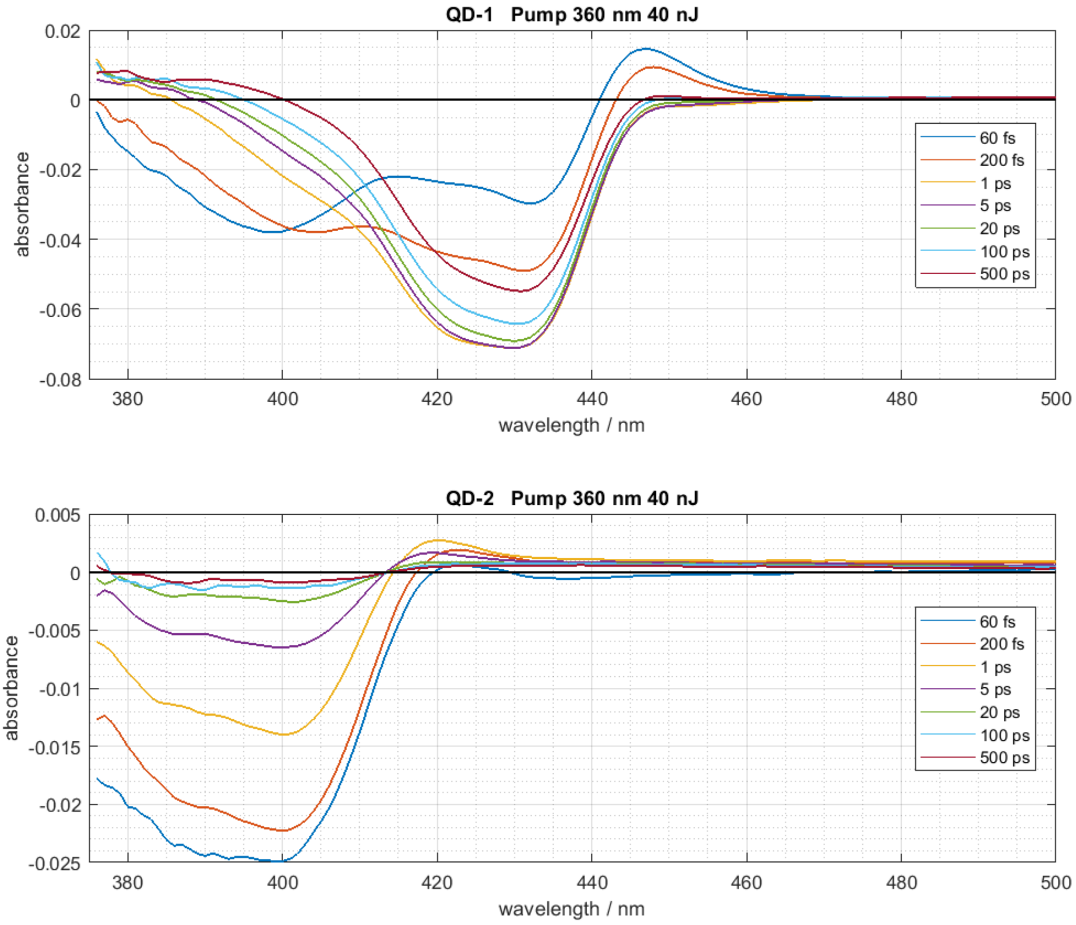

Figure S3. Femtosecond Pump-Probe TA spectra of QD-1 and QD-2 samples.

The TA spectra were measured with a femtosecond pumping probe using the supercontinuum method. The output of a titanium-sapphire oscillator (800 nm, 80 MHz, 80 fs, Tsunami, Spectra-Physics, USA) was amplified by a regenerative amplifier system (Spitfire, Spectra-Physics, USA). with a repetition rate of 1 kHz. The laser pulse frequency was controlled by a standard synchronization device and an SDG II Spitfire 9132 control amplifier manufactured by Spectra-Physics (USA). The device allowed changing the pulse repetition rate at the amplifier output from 0 to 1000 Hz. The amplified pulses were split into two beams. One of the beams was directed to an optical parametric amplifier with non-collinear phase matching. Its output, centered at 720 nm was transformed into second harmonics at 360 nm. The pump was a Gaussian pulse with a duration of 30 fs at a wavelength of 360 nm was used as the pump. The second beam was focused onto a thin quartz cuvette filled with H2O to generate supercontinuum probe pulses. The pumping and probing pulses were delayed in time relative to each other using a computer-controlled delay stage. They were then weakened, recombined, and focused on the sample cell. The pump and probe light spots had diameters of 300 and 120 μm, respectively.

The experiments were carried out at 293 K. The operating frequency of the pump pulse was 100 Hz, which is small enough to exclude permanent bleaching of the sample due to photochemical processes in the sample. The relative polarizations of the pump and probe beams were adjusted to 54.7 ° (magic angle) or parallel and perpendicular polarizations where indicated. After the sample, the supercontinuum was dispersed using a polychromator (Acton SP-300) and recorded using a CCD camera (Roper Scientific SPEC-10). Non-stationary spectra of changes in optical density ΔA (t, λ) were recorded in the range 380–850 nm. The dispersion of the supercontinuum group delay was measured from the

analysis of the laser artifact delay produced in the pure solvent according methods developed in using the procedure described by Kovalenko et al. [1]

[1] S.A. Kovalenko, A.L. Dobryakov, J. Ruthmann, N.P. Ernsting, Femtosecond spectroscopy of condensed phases with chirped supercontinuum probing, Phys. Rev. A - At. Mol. Opt. Phys. 59 (1999) 2369–2384. https://doi.org/10.1103/PhysRevA.59.2369.

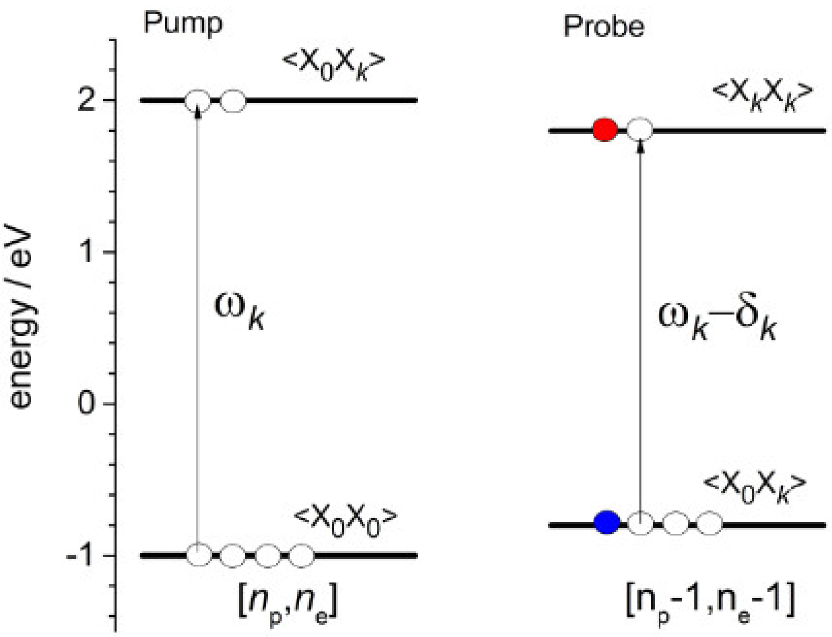

Figure S4. Decomposition of TA spectra into Gaussian peaks in the approximation of the model of exciton and biexciton transitions.

The intensity of the absorption band X_k in the linear spectrum is represented as the product

$$P_k = \sigma_k \cdot n_p \cdot n_e$$

$$\sigma_k \sim |\mu_k|^2$$

where σ_k is a cross section of optical transition, $\mu_k = \langle X_0 | \mu | X_k \rangle$ is the dipole moment of the transition to the X_k state, and n_p and n_e are the corresponding free electron and hole vacancies in the conduction band k. Excitation produces the configuration where some of these vacancies are filled; therefore, the intensity of the band decreases

$$P'_k = \sigma'_k \cdot (n_p - m) \cdot (n_e - m)$$

where m is multiplicity of the X_k exciton generated by the pump pulse. In the following we assume that the transition moment does not change upon excitation, i.e. $\mu'_k = \mu_k$, but the energy gap ω_k of the $X_0 \rightarrow X_k$ transition is altered due to exciton-exciton interactions δ_k. The change of energy gap δ_k due to multiexciton interactions is a special case of dipole polarization in an applied electric field (the Stark effect). The transient difference spectrum $\Delta S_k(\omega)$ of the X_k band is treated therefore as

$$\Delta S_k(\omega) \sim \sigma'_k \cdot (n_p - m) \cdot (n_e - m) \cdot G(\omega - \omega_k + \delta_k) - \sigma_k \cdot n_p \cdot n_e \cdot G(\omega - \omega_k)$$

where $G(\omega\text{-}\omega_k)$ is the Gaussian function

$$G_k(\omega - \omega_k) = exp(-(\omega - \omega_k)^2/2w_k^2)$$

The $\Delta S_k(\omega)$ spectrum includes two terms

$$\Delta S_k(\omega) = \Delta A_k^{Bl} \cdot G(\omega - \omega_k) + \Delta A_k^{St} \cdot [G(\omega - \omega_k + \delta_k) - G(\omega - \omega_k)]$$

The first term here is bleaching of the X_k band due to change in the n_p and n_e populations

$$\Delta A_k^{Bl} = -\sigma_k \cdot [m \cdot (n_p + n_e) - m^2]$$

and the second term is the Stark band shift

$$\Delta A_k^{St} = \sigma_k \cdot (n_p - m) \cdot (n_e - m)$$

Besides bleaching due to the changes of populations, there is also a contribution of stimulated emission with the band maximum shifted due to both Stokes and multiexciton interactions.
